# Prediction of Substrate-Enzyme-Product Interaction Based on Molecular Descriptors and Physicochemical Properties

**DOI:** 10.1155/2013/674215

**Published:** 2013-12-22

**Authors:** Bing Niu, Guohua Huang, Linfeng Zheng, Xueyuan Wang, Fuxue Chen, Yuhui Zhang, Tao Huang

**Affiliations:** ^1^Shanghai Key Laboratory of Bio-Energy Crops, School of Life Science, Shanghai University, 333 Nancheng Road, Shanghai 200444, China; ^2^Institute of Systems Biology, Shanghai University, Shanghai, China; ^3^Institute of Health Sciences, Shanghai Institutes for Biological Sciences, Shanghai 200444, China; ^4^Department of Radiology, First People's Hospital, Shanghai Jiaotong University, Shanghai 200080, China; ^5^Department of Neurosurgery, Changhai Hospital, Second Military Medical University, Shanghai 200433, China; ^6^Department of Genetics and Genomics Sciences, Icahn School of Medicine at Mount Sinai, New York, NY 10029, USA

## Abstract

It is important to correctly and efficiently predict the interaction of substrate-enzyme and to predict their product in metabolic pathway. In this work, a novel approach was introduced to encode substrate/product and enzyme molecules with molecular descriptors and physicochemical properties, respectively. Based on this encoding method, KNN was adopted to build the substrate-enzyme-product interaction network. After selecting the optimal features that are able to represent the main factors of substrate-enzyme-product interaction in our prediction, totally 160 features out of 290 features were attained which can be clustered into ten categories: elemental analysis, geometry, chemistry, amino acid composition, predicted secondary structure, hydrophobicity, polarizability, solvent accessibility, normalized van der Waals volume, and polarity. As a result, our predicting model achieved an MCC of 0.423 and an overall prediction accuracy of 89.1% for 10-fold cross-validation test.

## 1. Introduction

With the completion of gene sequencing projects, scientific focus is shifting from the investigation of the proteomics to metabonomics which is of chemical processes involving metabolites. Metabolism consists of almost all of the chemical-chemical reactions or chemical-macromolecules reactions that generally take place within metabolic pathway [1]. Above linked individual interactions form the whole metabolic pathway and interaction network which produce more new complex and higher order structure [2]. Metabolic pathways are sequences of metabolic steps forming highly regulated networks of interacting enzymes and substrates. In metabolic pathways, the substrate is transformed through a series of steps into another chemical, by a sequence of enzymes. Given a substrate and an enzyme, people may wonder whether they can interact with each other or what is the product. Herein, network of interaction of substrate-enzyme-product can provide assistance in R&D of drug. For example, based on interaction of substrate-enzyme-product, maybe people can discover some candidate drug from nature product, and can even predict its potential side effect [3]. Besides this, network of interaction of substrate-enzyme-product can also be applied in evaluating the safety of research of Genetically Modified Food (GMF). By using the network of substrate-enzyme-product, the potential toxicity of product derived from GMF could be predicted. Hence, the interaction network of substrate-enzyme-product will provide us further knowledge and information beyond metabolic pathway.

Due to the complexity of metabolic pathways, it is both time-consuming and costly to determine the interaction of substrate-enzyme-product by experiments. It is in urgent to develop a quick, reliable, and effective approach to predict the interactions among substrate, enzyme, and product.

In this study, we reported a computational approach for predicting the network of substrate-enzyme-product triads based on K-nearest neighbor (KNN) [4–6] algorithm combined with mRMR-IFS feature selection method.

## 2. Methods and Materials

### 2.1. Methods

#### 2.1.1. mRMR

Minimum Redundancy Maximum Relevance (mRMR), proposed by Peng et al., is an effective feature-selection method for evaluating the worth of an attribute by considering the minimum redundancy between attributes and the maximum relevance between attributes and targets [7]. More information of mRMR selection algorithm can be found in [7] and related studies [8–19].

#### 2.1.2. KNN

K-nearest neighbors (KNN) is the most basic instance-based machine learning technique classifying objects based on cluster theory [4–6]. KNN recognizes a sample's class according to the label on the K-nearest neighbors. The nearest neighbors of an instance are defined by the Euclidean distance [4]. KNN has been widely applied in the field of biological sciences [20–24]. More details about KNN can be referred to in [25, 26].

#### 2.1.3. Incremental Feature Selection (IFS)

First, construct *N* feature subset by incrementally adding features to *D* as follows:

(1)  D0={f0}, D1={f0,fi1},   ⋮ Di={f0,f1…,fi},   ⋮DN−1={f0,f1,…,fN−1}

(*f*_*i*_ is the *i*th feature added into feature subset *D*).

Second, use KNN method to build the prediction model based on subset *D*_*i*_ and evaluate the model by cross-validation. Then, a classification accuracy curve called IFS curve is attained.

### 2.2. Materials

#### 2.2.1. Data Preparation

In this study, 14,229 compounds derived from database KEGG (http://www.genome.jp/kegg/) (release 42 in 2006) [27] were collected. After removing the compounds which do not participate in any metabolic reactions which have been supported by experiments, 1326 compounds and 939 enzyme molecules of the human genome participating metabolic reaction were obtained (please refer to Supplemental Material available online at http://dx.doi.org/10.1155/2013/674215).

In metabolic pathway, each substrate binds to one or more enzymes, but the production may not be different. Therefore, substrates and enzymes are subject to be involved in a network of interactions. In this study, substrate, enzyme, and product in each interaction are defined as a positive sample; and those that cannot interact with each other or those interactions that cannot attain the product are defined as negative samples. Triads in the positive set are termed as networking triads, and those in the negative set as nonnetworking triads. These networking triads are supported by solid experiments with 100% credibility by KEGG. As a result, 14,592 networking triads were obtained. To generate the negative datasets, firstly, we built a dataset by randomly combining two small molecules and an enzyme together; then, we removed the 14,592 networking triads. It should be mentioned that although some nonnetworking triads may not be true nonnetworking triads by chance in negative database set, the chance is small. Therefore, the credibility of the negative dataset is also very high. To reflect that the number of networking triads is much less than that of the nonnetworking triads, the negative samples of training set were generated 50 times as many as the positive ones. As a result, the final training dataset contains 14,592 networking triads and 729,600 nonnetworking triads (please refer to supplemental material II and III for the data).

#### 2.2.2. Representation of Compounds

In developing a method for predicting drug-protein interaction, the first problem is how to describe this networking triad correctly as input for the prediction program. It is obvious that the performances of prediction model depend mostly on the features used to describe the molecular structures. In this study, molecular descriptors were applied to reflect the physicochemical and geometric properties of substrates and products which have been applied in our previous studies [28–30]. The values of these molecular descriptors were calculated by program ChemAxon which is available for computing the molecular descriptors [31, 32] (see supplemental material IV). As some molecular descriptors cannot be calculated for some compounds, finally totally 79 molecular descriptors are used in building the model. Before calculating molecule descriptors, the compounds' three-dimensional structures were optimized by using MM+ force field with the Polak-Ribiere algorithm until the root-mean-square gradient became less than 0.1 Kcal/mol. Then, the descriptors were calculated under stable conformation of each molecule based on AM1 semiempirical molecular orbital method at the restricted Hartree-Fock level with no configuration interaction.

#### 2.2.3. Representation of Enzymes

As each protein has its own physicochemical properties, like hydrophobicity, polarizability, and so vent accessibility, it is a good method to describe a protein sequence, and it has been employed for predicting various protein attributes. In this paper, the enzymes are encoded by 132 physicochemical descriptors (amino acid composition, predicted secondary structure, hydrophobicity, polarizability, solvent accessibility, normalized van der Waals volume, and polarity) [33–38] (see supplemental material V) due to its effective and selective ability in the prediction of protein characteristics. More details can be seen in reference [33–38] or our previous study [39].

### 2.3. Accuracy Measure

Generally speaking, the prediction performance of different discriminative methods is commonly evaluated by the function of true positives (TP), true negatives (TN), false positives (FP), and false negatives (FN). In this study, we employed sensitivity (SN = TP/[TP + FN]), specificity (SP = TN/[TN + FP]), overall accuracy (ACC = [TP + TN]/[TP + TN + FP + FN]), and Matthew's correlation coefficient (MCC) to measure the prediction. The MCC can be represented as

(2) MCC =TP×TN−FP×FN(TN+FN)×(TN+FP)×(TP+FN)×(TP+FP).

## 3. Results

In the recent years, many efforts have been made in feature selection [40–46]. In this study, mRMR method was applied to search for a subset with optimal features. After mRMR calculation, two tables are attained (see supplemental material VI). One is called MaxRel feature table that ranks the features based on their relevance to the class of samples and the other is called mRMR feature table that lists the ranked features by the maximum relevance and minimum redundancy to the class of samples.

**Figure 1:**
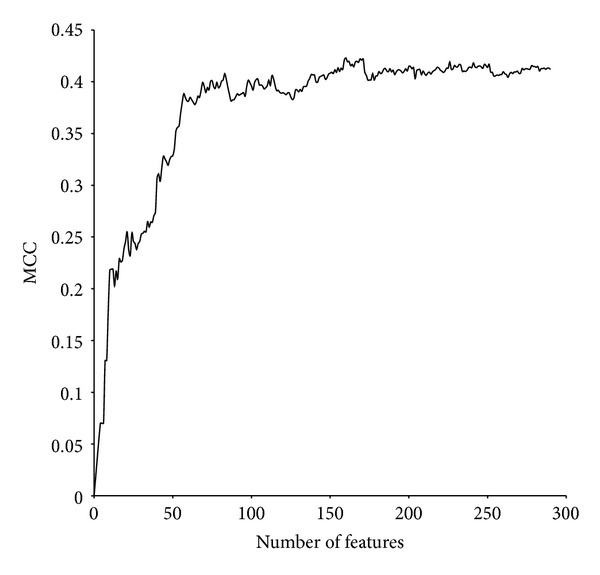
The curve of the 290 prediction models using IFS.

Then, IFS method is applied based on mRMR feature table. From Figure 1, it can be found that while adding new feature continually, the value of MCC increased, although during this process, the value of MCC decreased at some point. While the number of features reaches 160, the value of MCC is 0.423, the highest point. Then, the value of MCC begins to decrease. Hence, the subset containing these 160 features is considered as an optimal subset which is derived from original data set containing 290 features. These features selected are irrelevant to each other but relevant to the target.

Based on the 160 features, predicting model of network of substrate-enzyme-production interaction could be built.

Ten folds cross-validation test, which is applied in many other applications [36, 47–52], is adopted in this study to validate the model's prediction accuracy. During 10-fold cross-validation test, the datasets are divided into 10-folds, a model is built with N-1 fold samples and the 10th fold data are treated as unseen data, which is used for the prediction as the testing data. Each fold is left out from building the model and predicted in turn. The predictive ability is evaluated by averaging the correct prediction rates of the 10-fold data.  Table 1 lists the prediction results while using KNN method.

To evaluate our feature selection method, we compared the prediction results generated by final optimal subset and the original data set with 10-folds cross validation test (see Table 1).  Table 1 shows that the prediction results of the 10-folds cross-validation test improved after applying feature selection. This demonstrates that maybe some features are redundant and interfering to each other in the original dataset; hence, it is better to remove some of them. Furthermore, the number of features in the final subsets is 55% of the original feature set. This result suggests that mRMR feature selection approach could make a good optimization and improve the accuracy of prediction for substrate-enzyme-product interaction.

## 4. Discussion

**Figure 2:**
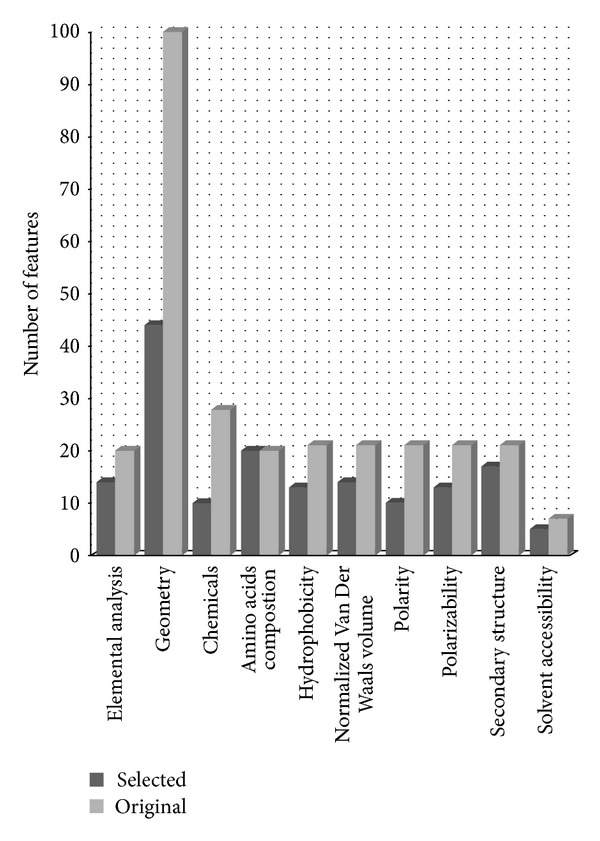
Feature distribution.

The selected 160 features in the final subset can be clustered into the following ten categories: elemental analysis, geometry, chemistry, amino acid composition, predicted secondary structure, hydrophobicity, polarizability, solvent accessibility, normalized van der Waals volume, and polarity (see Figure 2). The former three kind features are molecular descriptors which are of substrate and product, and the left seven kind features are of enzyme.

According to the distribution of features of compounds (substrate and product) and enzymes, it shows that enzymes contribute more to the interaction process. Further calculating the proposition of the selected features to the original features, it is found that the proposition of enzyme feature (92/132 = 0.70) is higher than the proposition of compound feature (70/158 = 0.44).  Table 2 also shows that several enzyme features are in the top ten and top twenty features. This result suggests that enzyme-centric features make more contributions to our proposed interactions network of substrate-enzyme-product.

From Table 2, it can be further found that for compound features, there are much less features of product than features of substrate and enzyme in the top fifty features. This is because during the interaction of substrate-enzyme-product, substrate and enzyme determine the products, and changing substrate or enzyme could result in a different product.

According to the distribution of features in Figure 2, it can be found that the number of geometry features is more than that of the other kind features. In this regard, geometry features have great effect and contribute to the substrate-enzyme-product interaction not only in substrate features but also in product features. However, from MaxRel feature table, it can be found that there are not many geometry features appearing in the top ten features. Therefore, we feel interesting of this problem. Actually, the order of geometry features is not incompatible with its distribution. Geometry features contain information of the structure of a molecule like the volume, size, and shape which leads to steric hindrance and steric resistance. These factors are of great importance in substrate-enzyme-product interaction. Only correctly three-dimensional size and shape molecule can interact with enzyme according to the Lock and Key Theory. Meanwhile, steric hindrance or steric resistance affect the substrate-enzyme-products' interaction as some big functional groups like aromatic ring prevent interaction. On the other hand, these functional groups also provide key interactive force to enzyme like heteroaromatics ring's *π*-*π* stacking interaction to enzyme's functional site. The substrates and products are varied and diverse greatly in structure. And it is difficult to describe their structure with only one or two descriptors. Hence, more geometry features could better extract the information of compounds' structure. This is why though single geometry feature has no strong relevance to the interaction, the overall contribution of the forty-four geometry feature can often be crucial to the interaction.

Figure 2 also shows that amino acid compositions and second structure occupied important propositions among the ten types' features. Amino acid composition in the binding site contributes a lot in substrate-enzyme-product interaction because it could affect the state energy. Some experiments have verified the importance for amino acid compositions in protein related interaction [53–55]. For example, Tyr265 plays a central role in enzyme alanine racemase's binding to L-alanine and pyridoxal 5-phosphate [54]. Hence, for a unique structure, the amino acid composition plays the essential role in the interactions. Secondary structure is considered as an important property in many protein related problems, since the shape and biological function of a protein are mainly determined by its secondary structures. Secondary structure features reflect the steric structure of protein. According to the Lock and Key Theory, the size and shape of substrate were rigid and restricted by enzyme. Accordingly, secondary structure has relatively more impact on the determination of substrate and product.

## 5. Conclusion

In this paper, a feature selection method called mRMR combined with IFS was applied to dataset of substrate-enzyme-product interaction which is encoded with molecular descriptors of substrate/product and 132 physicochemical protein descriptors. As a result, we find that enzymes are essential in substrate-enzyme-product interaction; 160 important features were abstracted from 290 features. Based on the above findings, we also used KNN method to build a prediction model of substrate-enzyme product interaction. Based on the prediction results, it is expected that molecular descriptors and 132 physicochemical protein descriptors can be served as an efficient coding method for network of substrate-enzyme-product interaction.

## Supplementary Material

Supplementary Material 1*：* The ID of 1326 compounds and 939 enzymes of metabolic pathway in Kegg database.
Supplementary Material 2*：*14,592 networking triads of substrate-enzyme-product.
Supplementary Material 3: 729,600 non-networking triads of substrate-enzyme-product.
Supplementary Material 4: Calculated value of molecular descriptors of 1326 compounds and the categories.
Supplementary Material 5: The value of 132 physicochemical descriptors for 939 enzyme.
Supplementary Material 6: The MaxRel and mRMR feature tables using mRMR method.

## Figures and Tables

**Table 1:** Prediction accuracies of different dataset with KNN.

Dataset	10-folds cross-validation test			
	SN (%)	SP (%)	ACC (%)	MCC
Original dataset	53.71	92.4	88.9	0.412
Optimal dataset	55.2	92.4	89.1	0.423

**Table 2:** Top 80 features rank according to their correlation to target.

No.	Name	Categories	No.	Name	Categories
1	Polarity	Polarity	41	Amino Acids Composition Cys	Amino acids composition
2	Substrate_ Polarizability	Chemical	42	Polarizability	Polarizability
3	Solvent accessibility	Solvent accessibility	43	Polarizability	Polarizability
4	Solvent accessibility	Solvent accessibility	44	Amino Acids Composition Ile	Amino acids composition
5	Secondary structure	Secondary structure	45	Hydrophobicity	Hydrophobicity
6	Normalized Van Der Waals volume	Normalized Van Der Waals volume	46	Secondary structure	Secondary structure
7	Normalized Van Der Waals volume	Normalized Van Der Waals volume	47	Substrate_Stereo DoubleBondCount	Geometry
8	Secondary structure	Secondary structure	48	Normalized Van Der Waals volume	Normalized Van Der Waals volume
9	Secondary structure	Secondary structure	49	Substrate_Smallest RingSystemSize	Geometry
10	Substrate_ Log*P*	Chemical	50	Substrate_Smallest RingSize	Geometry
11	Substrate_ CComposition	Elemental analysis	51	Substrate_Rotatable BondCount	Geometry
12	Amino Acids Composition Asn	Amino acids composition	52	Substrate_H Composition	Elemental analysis
13	Polarity	Polarity	53	Amino Acids Composition Thr	Amino acids composition
14	Hydrophobicity	Hydrophobicity	54	Polarizability	Polarizability
15	Substrate_MinZ	Geometry	55	Amino Acids Composition Leu	Amino acids composition
16	Solvent accessibility	Solvent accessibility	56	Amino Acids Composition His	Amino acids composition
17	Polarity	Polarity	57	Substrate_CarboAliphatic RingCount	Geometry
18	Hydrophobicity	Hydrophobicity	58	Product_HComposition	Elemental analysis
19	Substrate_VanDerWaals SurfaceArea	Chemical	59	Polarizability	Polarizability
20	Amino Acids Composition Asp	Amino acids composition	60	Normalized Van Der Waals volume	Normalized Van Der Waals volume
21	Hydrophobicity	Chemical	61	Amino Acids Composition Gln	Amino acids composition
22	Substrate_ OComposition	Elemental analysis	62	Normalized Van Der Waals volume	Normalized Van Der Waals volume
23	Solvent accessibility	Solvent accessibility	63	Polarizability	Polarizability
24	Secondary structure	Secondary structure	64	Amino Acids Composition Lys	Amino acids Composition
25	Amino Acids Composition Ser	Amino acids composition	65	Polarizability	Polarizability
26	Substrate_Water AccessibleSurface Area Negative	Chemical	66	Amino Acids Composition Tyr	Amino acids composition
27	Secondary structure	Secondary structure	67	Amino Acids Composition Arg	Amino acids composition
28	Hydrophobicity	Hydrophobicity	68	Secondary structure	Secondary structure
29	Substrate_FusedRingCount	Geometry	69	Polarizability	Polarizability
30	Substrate_Carbo RingCount	Geometry	70	Normalized Van Der Waals volume	Normalized Van Der Waals volume
31	Amino Acids Composition Glu	Amino acids composition	71	Polarity	Polarity
32	Hydrophobicity	Hydrophobicity	72	Normalized Van Der Waals volume	Normalized Van Der Waals volume
33	Polarizability	Polarizability	73	Product_NComposition	Elemental analysis
34	Polarity	Polarity	74	Solvent accessibility	Solvent accessibility
35	Normalized Van Der Waals volume	Normalized Van Der Waals volume	75	Product_Hetero AliphaticRingCount	Geometry
36	Substrate_Fused Aliphatic RingCount	Geometry	76	Substrate_CarboAromatic RingCount	Geometry
37	Polarizability	Polarizability	77	Substrate_PComposition	Elemental analysis
38	Secondary structure	Secondary structure	78	Hydrophobicity	Hydrophobicity
39	Substrate_RingCount	Geometry	79	Product_CComposition	Elemental analysis
40	Amino Acids Composition Pro	Amino acids composition	80	Normalized Van Der Waals volume	Normalized Van Der Waals volume
